# Rapid adsorption of selenium removal using iron manganese-based micro adsorbent

**DOI:** 10.1038/s41598-022-21275-4

**Published:** 2022-10-14

**Authors:** Sundus Saeed Qureshi, Sheeraz Ahmed Memon, Rafi-ul-Zaman, Nanik Ram, Sumbul Saeed, Nabisab Mujawar Mubarak, Rama Rao Karri

**Affiliations:** 1 Institute of Environmental Engineering and Management, Mehran University of Engineering and Technology, Jamshoro, 76090 Sindh Pakistan; 2 College of Plant Sciences and Technology, Huazhong Agricultural University, Wuhan, 430070 People’s Republic of China; 3 Petroleum and Chemical Engineering, Faculty of Engineering, Universiti Teknologi Brunei, Bandar Seri Begawan, 1410 Brunei Darussalam

**Keywords:** Environmental sciences, Environmental social sciences, Energy science and technology, Materials science, Nanoscience and technology

## Abstract

Selenium in wastewater is of particular concern due to its increasing concentration, high mobility in water, and toxicity to organisms; therefore, this study was carried out to determine the removal efficiency of selenium using iron and manganese-based bimetallic micro-composite adsorbents. The bimetallic micro-composite adsorbent was synthesized by using the chemical reduction method. Micro-particles were characterized by using energy-dispersive X-ray spectroscopy for elemental analysis after adsorption, which confirms the adsorption of selenium on the surface of the micro-composite adsorbent, scanning electron microscopy, which shows particles are circular in shape and irregular in size, Brunauer–Emmett–Teller which results from the total surface area of particles were 59.345m^2^/g, Zeta particle size, which results from average particles size were 39.8 nm. Then it was applied to remove selenium ions in an aqueous system. The data revealed that the optimum conditions for the highest removal (95.6%) of selenium were observed at pH 8.5, adsorbent dosage of 25 mg, and contact time of 60 min, respectively, with the initial concentration of 1 ppm. The Langmuir and Freundlich isotherm models match the experimental data very well. The results proved that bimetallic micro-composite could be used as an effective selenium adsorbent due to the high adsorption capacity and the short adsorption time needed to achieve equilibrium. Regarding the reusability of bimetallic absorbent, the adsorption and desorption percentages decreased from 50 to 45% and from 56 to 53%, respectively, from the 1st to the 3rd cycle.

## Introduction

Around 71% of drinking water in Pakistan is accessed from underground aquifer reservoirs. From the recently developed water policy 2018, the Pakistan government aims to provide filtered drinking water to all the occupants of Pakistan by 2025 by controlling the delivered toxins in water and by treating water to restrict water-borne diseases. The water whack in Pakistan decreased in 1952 from 5000 m^3^ to 1100 m^3^, which shows that country will face the water scarcity problem overall. The study estimates that water per capita will be around 700 m^3^ or even less by 2025^1^. An improper and misuse of water resources have deteriorated the quality and quantity of groundwater^2^. A national-level study shows that human activities such as increased mining activities and groundwater pits in petroleum industries tend to increase water contamination in underground water resources, as reported by the united nation world water development report in 2015^3^.

Selenium has been widely found in different industries such as electronic, chemical, pharmaceutical, ceramic, metallurgy, paint industry, etc. Subsequently, selenium may go downstream of surface water, plants, and soil through the high effluent generated by these industries^4^. The presence of selenium in the aquatic environment is caused naturally and synthetically. Several natural sources cause the presence of selenium in water, such as weathering of soil and rock, volcanic eruption, and chemical inter-conversion in coal mines. selenium was found in water due to man-made activities: the coal combustion industry, agricultural activities, mining, oil and gas refining process, pesticide production, glass, and cement industries. During power generation, solid waste and flue gases are released into the environment, which contributes high concentration of selenium in water in the form of SeO_2_ and SeO gases^5^. It has been estimated that anthropogenic activities are responsible globally for releasing 76,000–88,000 tons of selenium per year into the soil and water, which is then transferred to animal organisms, plants, and the life cycle, resulting in serious environmental and health effects^6,7^. According to guidelines by the European Union, National Standards for Drinking Water Quality (NSDWQ) and World Health Organization (WHO), selenium uptake in drinking water is 10 μg/L^8–10^. Therefore, it is required to remove selenium from groundwater to the standard level.

Various techniques have been used to remove selenium from water, including reverse osmosis and chemical reduction, coagulation, membrane filtration, bacterial reduction, and ion exchange^11,12^. However, the presence of toxic chemicals, high operating system, and cost hinder the applications of these techniques in a user-friendly way^13–16^. The adsorption technique is assumed to be an alternative technique for the remediation of selenium from water. Over the past decades, adsorption has been broadly utilized due to the greatest opportunity for sustainable implementation for the ease of reusability and the production of adsorbing media^17–24^. The water treatment based on using adsorbent material to remove the contaminants from the aqueous medium is considered eco-friendly and low-cost for remediation of selenium, thus cleaning and purifying the groundwater, drinking water, and industrial wastewater^18,25–36^. The regeneration/reuse is one of the advantages of the conjugate adsorbent, which can be recycled to elute the Se (IV) from the adsorbent for reuse in water treatment. To evaluate the regeneration capability of the adsorbent, we have defined the suitable eluent for complete elution and regeneration without loss in its cage cavities and reusability of adsorbent^37^. The disadvantage is that they produce large amounts of toxic sludges that require further treatment before disposal into the environment^38^. The Fe or Mn compounds are generally safe for humans and the environment, but there are still some of them like nZVI can be toxic^39^.

Several metal oxides are used as efficient adsorbents for scavenging pathways for many heavy-metal ion removals^40^. Adsorption of selenium by different bimetallic compounds as adsorbents is shown in Table 1. Different literature and case studies suggest that the adsorption by bimetallic micro-particle is the most advanced technology compared to other technologies for removing selenium from water. In the previous literature, various adsorbents for wastewater treatment removal have been utilized, including cotton husk carbon material, activated carbons, a high-molecular polymer, and transition metal oxides (Al_2_O_3_, Fe_3_O_4_, MnO_2_, TiO_2_)^41–44^. Due to the high-affinity interaction between selenium and iron possessing the outstanding magnetic properties of the iron material, iron-based materials such as the iron-manganese bimetallic composites are considered the best adsorbents for water treatment^45–49^. A binary oxide of Fe–Mn showed characteristics of high adsorption power for (iron oxides), high oxidation efficiency for (manganese dioxide), and outstanding separating efficiency for (magnetic materials)^50–52^. Therefore, it is highly urged to develop an adsorbent with the characteristics features of strong adsorption efficiency, significant adsorption mechanism through characterization, strong adsorption ability, and adsorption kinetics and isotherms, respectively^53–57^.

**Table 1:** Adsorption of selenium by different bimetallic compounds as adsorbents.

Bi-metallic compounds	Selenium removal	Isotherms model	Time	pH	Removal efficiency	References
zirconium and iron oxides	Se(IV)	Langmuir model	–	8	90	^58^
Mg–FeCO_3_	Selenite	–	–	6	92	^59^
MNP@hematite	Se(IV)	–	10 min	4 to 9	97	^60^
Iron oxide impregnated CNTs	selenium	Langmuir model		6	111	^61^
Iron oxide nanoparticle	Selenium	–	–	4	95–98	^62^
Chitosan–clay composite	Selenium	Langmuir model	–	4	18.4	^63^
Iron-coated GAC	Selenite	Langmuir model		2–8	97.3	^22^
Nanocrystalline hydroxyapatite	Se (IV)	Freundlich isotherm	90 min	5	1.94	^21^
Aluminum oxide-coated sand	Se (IV), Se (VI)	Langmuir model	60 min	4.80	1.08	^27^
Sulfuric acid-treated rice husk	Se (IV)	Langmuir model	–	1.5	40.92	^30^

Therefore, this study mainly aims to synthesize a novel iron-manganese-based adsorbent to remove selenium. In addition of effect of process parameters such as pH, dosage, time and concentration were optimized. Furthermore, the isotherm and kinetics are applied for the removal of selenium. Moreover, the iron-manganese composite is characterized by EDX analysis, XRD analysis, SEM analysis, zeta potential analysis, BET analysis.

## Material and methods

### Materials

Selenium (IV) Oxide (SeO_2_) (Mw = 110.96 g), Iron (II) Chloride Hexahydrate, Manganese Chloride, Sodium Tetrahydridoborate 97^+^%, Hydrochloric Acid, and Sodium Hydroxide was purchased from Sigma Aldrich (USA). All the synthetic substances utilized were of the analytical grade.

### Synthesis of iron-manganese (Fe–Mn) bimetallic micro-particles

The micro-composite adsorbent was synthesized by the chemical reduction method by making two separate solutions, i.e., iron and manganese solution and the sodium tetraborate solution. The solution of equimolar concentration (w/w) of Fe and Mn was prepared separately by dissolving 11.30 mg iron chloride salt in 50 ml (0.1 M) and 11.45 mg of manganese chloride in 50 ml distilled water. Both salts were then mixed to make 100 mL of solution. Also, the sodium tetraborate solution was prepared by adding 200 mg of sodium tetraborate to 50 mL of arrangement. The arrangement of salts was set on the magnetic stirrer for proper mixing. At the same time, a sodium tetraborate solution was added dropwise into the salt solution to prepare microparticles. The addition of sodium tetraborate was continued until the solution became dark brownish, indicating the formation of microparticles. Then the solution was filtered using wattman filter paper, and the particles were collected separately. Collected particles were dried in the oven at 50 °C for 24 h.

### Characterization of bimetallic adsorbent

The synthesized micro-particles were characterized by scanning electron microscopy (SEM), energy-dispersive X-ray spectroscopy (EDX), Brunauer–Emmett–Teller (BET), and zeta particle size, respectively. SEM analysis of the samples was conducted by FEI Quanta Scanning Electron Microscope (Thermo Fisher Scientific, United States) to identify the surface changes of the samples. All samples were iridium coated. The experiment was run under a high vacuum environment. The Malvern Zetasizer Nanozs, UK, analyzed particle size and surface charge. 0.01 g of each sample was dispersed into 1 mL and 0.75 mL of water separately into the different vessels for particle size and zeta potential (surface charge). Both cuvettes were inserted into the zeta sizer machine and analyzed the results through Malvern software connected with the zeta sizer. The Brunauer–Emmett–Teller (BET) analysis and Brookhurst Junior High (BJH) method determined the samples' surface area and porosity analysis. N_2_ adsorption/desorption isotherms, surface area, pore diameter, and pore volume were obtained at 77.25 K using Micro metrics TriStar II 3020 version 3.02 (ATA Scientific Pty Ltd., Australia). All samples were degassed at 120 °C under vacuum for 18 h before BET analysis.

### Preparation of selenium stock solution

The standard stock selenium solution was prepared by adding 140 mg of selenium Dioxide into 100 mL of distilled water to prepare 1000 ppm of selenium solution. The concentration of selenium solution was quantified using a hydride generation atomic absorption spectrophotometer (AAS). Moreover, to prepare a variety of various smaller concentrations of selenium solution, such as 0.1 ppm, 0.5 ppm, 1 ppm, 2 ppm, 5 ppm, and 10 ppm, the dilution was done from the stock solution to carry out batch experiments.

### Batch adsorption experiments

To decide the advanced boundaries for removing selenium from water, the Batch tests were completed on a 10-ppm focus. Different concentrated selenium solution (0.1 ppm, 0.5 ppm, 1 ppm, 5 ppm, 8 ppm, 10 ppm) solutions with a working volume of 50 ml were made in an adjusted base carafe with the weighted measure of 25 mg of adsorbent of bimetallic micro-particles and rotated in the stir at 100 rpm for 1 h on a shaker. Adsorbent portion, shaking time, Adsorption Concentration, and impact of pH was explored. The rest of the selenium concentrations were measured on AAS. Moreover, to determine the selenium concentration using a hydride generation atomic absorption spectrophotometer, the selenium is first converted into hydrogen selenide SeH_2,_ then decomposed in a heated cuvette. To reduce selenium solution into hydrogen selenide, sodium borohydride NaBH_4_ is commonly used^64^.

Selenium stock solution with the convergence of 1000 mg/L was made, and another diverse focus was acquired by the ceaseless weakening of 0.1 M arrangement of NaOH and 0.1 M arrangement of HCl to change the distinctive pH of the arrangements. All the readings were completed in 50 ml arrangements for the selenium removal from water. Group tests were completed by taking the weighted 25 mg of bimetallic NPs in 50 mL arrangements containing 10 ppm of selenium focus. The parameters affecting selenium adsorption were optimized by transforming one factor at a solitary time and keeping the others the same. The impact of time was inspected by shaking the selenium arrangement and bimetallic NPs blending at 100 rpm. The impact of shaking time on the efficiency of selenium removal by bimetallic micro-composite adsorbent was studied by taking all the other parameters constant with a solution of the initial concentration of 10 ppm, 0.5 g/L of adsorbent dosage at 6.5 pH of the solution by varying time from 15 to 120 min. In driving the adsorption process, the pH of the solution is an important parameter. This study examines the pH effect on selenium removal from the water. To determine the pH value on removal efficiency of selenium by the bimetallic micro-composite adsorbent, samples were prepared at different pH values, i.e., 6.5, 7, 7.5, 8, 8.5, 9, with a solution of a beginning grouping of 10 ppm, adsorbent dose of 0.5 g/L, at 60 min of adsorption time. The optimum condition for the dosage was selected by micro-particle mass from 5 to 30 mg. The selenium removal efficiency was studied on six different dosages from 5 to 30 mg while keeping the range of various boundaries steady, i.e., 10 ppm selenium arrangement at pH 6.5 fomented for 1 h for finding the selenium removal efficiency and removal limit of bimetallic micro-composite adsorbent. The impact of initial concentration was studied by taking selenium concentrations from 0.1 to 10 mg/L.

Furthermore, the final concentrations were noted after the treatment of the sample with Bimetallic MPs. The formula calculated the removal percentage of selenium:

1 $$Removal \%= \frac{{C}_{i}-{C}_{f}}{{C}_{i}} \times 100$$

Cf and Ci are final and initial selenium concentrations in mg/L, respectively.

The percent adsorption was calculated by using the following equation:

2 $${Q}_{e}= \frac{{C}_{o}-{C}_{e}}{m}\times V$$

where: Q_e_ = removal capacity (mg/g), C_o_ = initial concentration of Se in solution (mg/L), C_e_ = final concentration of Se in solution after contact with the bimetallic adsorbent (mg/L), V = Volume of the arrangement (L), m = Mass of nano particles (g).

### Isotherm studies

The equilibrium data obtained from the experiments were fitted to different isotherm models to understand the inherent mechanisms in the selenium adsorption on Fe–Mn Bimetallic micro-composite. The conventional solid–liquid adsorption isotherm models (single component) evaluated in this study are presented in Table 2. This table shows non-linear forms of isotherm models. It is a common practice by most researchers to linearize the non-linear model expressions, as it is not straightforward to calculate constants in the non-linear models. Even though the linearized model equation adequately fits the equilibrium data, providing higher R^2^, the predicted values gave low values after the isotherm parameters were substituted in the non-linear model form.

Further, to statistically validate the performance of predicted values with the experiments, various prominent statistical (error) functions were chosen, as shown in Table 3. These metrics confirm and validate the suitability of respective non-linear isotherm models and evaluated parameters^65,66^. For instance, if the metrics result in a smaller value (with the exception of R^2^) this means that the model-predicted values are in close agreement with the experimental values; however, they vary distinctly if it produces a higher metrics results.

**Table 2:** Different prominent isotherm models (along with a number of parameters they have in respective models) that were investigated in Selenium adsorption on Fe–Mn Bimetallic micro-composite.

Isotherm and kinetic models	Non-linear equations	Number of parameters
**Isotherm models**		
Linear (Henry's Law) model	$q_{e} = KC_{e}$	1
Langmuir model	$q_{e} = \left( {\frac{{K_{L} bC_{e} }}{{1 + bC_{e} }}} \right)$	2
Freundlich model	$q_{e} = K_{F} C_{e}^{1/n}$	2
Redlich-Peterson model	$q_{e} = \left( {\frac{{K_{R} C_{e} }}{{1 + a_{R} C_{e}^{\alpha } }}} \right)$	3
Sips model	$q_{e} = \left( {\frac{{K_{s} bC_{e}^{1/n} }}{{1 + bC_{e}^{1/n} }}} \right)$	3
Toth model	$q_{e} = \frac{{K_{th} C_{e} }}{{\left( {b^{\prime} + C_{e}^{n} } \right)^{1/n} }}$	3

Ce (mg/L) is the amount at equilibrium, qe (mg/g) is the measure of adsorbate adsorbed with a unit weight of adsorbate.

**Table 3:** Different prominent statistical (error) functions evaluated in this study to validate the performance of non-linear model prediction.

Statistical (error) functions
$$\text{Coefficient of correlation }({R}^{2})=\frac{{\sum }_{i=1}^{n}\left[{\left({q}_{e,pred}^{i}-{q}_{e,exp}^{i}\right)}^{2}\right]}{{\sum }_{i=1}^{n}\left[{\left({q}_{e,pred}^{i}-{\overline{q} }_{e,exp}^{i}\right)}^{2}\right]}$$
$$\text{Sum of absolute error }(SSE)={\sum }_{i=1}^{n}\left({q}_{e,pred}^{i}-{q}_{e,\mathrm{exp}}^{i}\right)$$
$${\text{Pearson}}^{\prime}s\text{ Chi-square measure}({\chi }^{2})={\sum }_{i=1}^{n}\frac{{(q}_{e, pred}^{i}-{q}_{e,\mathrm{exp}}^{i}{)}^{2}}{{q}_{e, pred}^{i}}$$
$$\text{Root mean square error }(RMSE)=\sqrt{\frac{1}{n-1}{\sum }_{i=1}^{n}{(q}_{e, pred}^{i}-{q}_{e,\mathrm{exp}}^{i}{)}^{2}}$$
$$\text{Hybrid fraction error function }(HYBRID)=\frac{100}{(p-n)}{\sum }_{i=1}^{p}\frac{{(q}_{e, pred}^{i}-{q}_{e,\mathrm{exp}}^{i}{)}^{2}}{({q}_{e,pred}^{i}{)}^{2}}$$

where ‘*n*’ is the number of experimental runs and ‘*p*’ is the number of parameters; ${q}_{e,exp}^{i}$ is q_e_ (mg/L) experiment value for each run; ${q}_{e,pred}^{i}$ is q_e_ (mg/L) predicted (calculated) value for each run and ${\overline{q}}_{e,exp}^{i}$ is mean of all q_e,exp_ (mg/L) values.

### Reusability of microparticles

To determine the reusability of micro-particles, the adsorption study was carried out in the same way, to achieve better selenium absorption on the micro-particle surface. After selenium adsorption, a desorption study was investigated by drying the used micro-particles in the oven at 100 °C to remove water from the micro-particles. Dried micro-particles were collected and mixed in 50 mL of 6 pH distilled water and stirred at 100 rpm for 60 min on a Rotary Shaker (Wisd. Laboratory Instruments) to resuspend the adsorbed selenium and achieve desorption. The desorption efficiency was calculated using the following equation.

3 $$Desorption\,percentage \%= \frac{Concentration\,desorbed}{Concentration\,absorbed}\times 100$$

Their consecutive desorption/ adsorption cycles were performed to determine the reusability of bimetallic micro-particles. The removal efficiency of each cycle was calculated by following the equation.

4 $$Removal\,percentage \%= \frac{Concentration\,absorbed }{Initial\,concentration}\times 100$$

## Results and discussion

### Characterization of bimetallic nanoparticles

#### Scanning electron microscope (SEM) analysis

**Figure 1:**
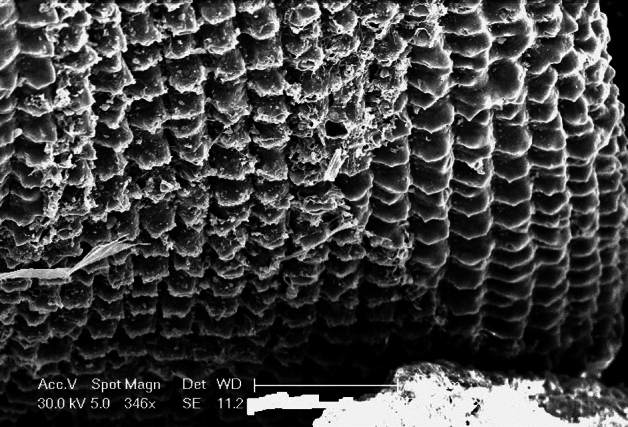
SEM image of bimetallic micro adsorbent.

SEM analysis is one of the most widely used techniques to characterize micromaterials and microstructures. Scanning electron microscopy was used to observe the morphological analysis of the Fe–Mn-based bimetallic micro-composite adsorbent, including shape, size, and porosity. The morphology of raw bimetallic micro-composite adsorbent before adsorption is shown in Fig. 1. The SEM images reveal that microparticles have been synthesized successfully and have the fine granular structure been spherical. The structure of the Fe–Mn-based bimetallic micro-particles was porous and spherical. The microporous structure is beneficial for the internal diffusion of adsorbents to remove heavy metal ions^67^. Previous studies reported that the SEM of Fe–Mn adsorbent showed that the material was constituted by many aggregated small particles, leading to a rough surface and porous structure^51^. Another study reported that uniform pore sizes into large particle sizes are advantageous for a suitable substrate for organic ligand immobilization^37^.

#### EDX analysis

**Figure 2:**
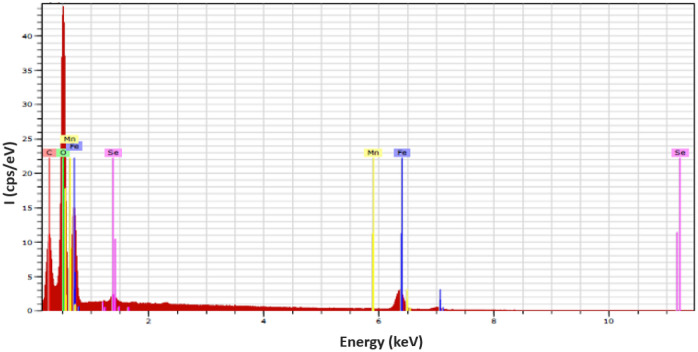
EDX Spectrum analysis of iron-based manganese composite.

Energy-dispersive X-ray spectroscopy (EDX) is an analytical technique used for elemental analysis; therefore, elemental analysis of Fe–Mn bimetallic micro-particles was examined using EDX analysis, which proved to be a very convenient method for analyzing this material. Elemental analysis of the particles was carried out to confirm selenium adsorption on the particles after use. The typical EDX spectrum of bimetallic micro-particles is shown in Fig. 2. The strong peak in the EDX spectra indicates the presence of selenium at 1.5 keV. This result demonstrates the high purity of the Fe–Mn bimetallic micro-particles. Similar analyses were reported by another researcher in 2019^68^. After the synthesis of Fe–Mn oxide, the elemental analysis reported in previous studies showed that the iron is distributed quite homogeneously within the particles. However, the absolute manganese content showed a slight gradient in the core of particles^69^. The elemental composition of iron-manganese-based composite by EDX analysis suggests that both iron and manganese were demonstrated at the intense signal of 0.5 keV and 6 keV; however, manganese was a major element as compared to iron. Additionally, the intensity range of 1.5 keV and 10.5 keV shows the presence of selenium. The minor signals were also found in the range of 0.5-1 keV, which represent the availability of carbon and oxygen elements.

#### BET analysis

**Figure 3:**
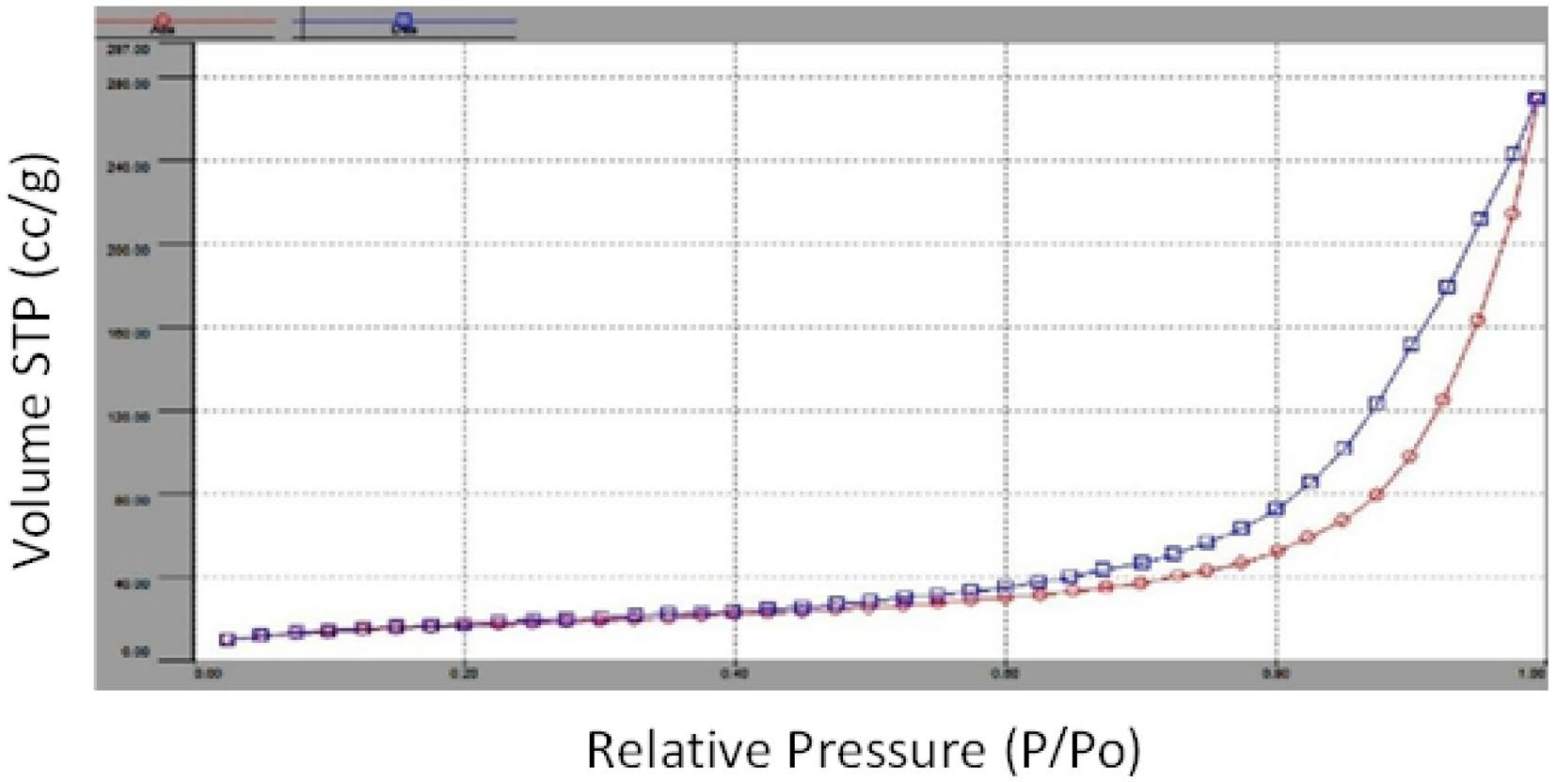
BET Analysis of iron-based manganese composite.

Brunauer–Emmett–Teller (BET) analysis explains the physical adsorption of gas molecules on a solid surface. It serves as the basis for an important analysis technique for measuring the specific surface area of materials. The surface area of the Fe–Mn bimetallic micro-particles was found to be 59.345 m^2^/g, as shown in Fig. 3. The higher surface area obtained in this study indicates that Fe–Mn bimetallic micro-particles have a porous structure which ultimately provides high adsorption capacity, indicating a good possibility of the material to be used as a surface adsorber. The surface area of adsorbents is one of the most significant properties in determining absorbance efficiency. A higher surface area enhances the adsorption capacity. The surface area of the experimented samples in this study was determined by the nitrogen (N_2_) adsorption method. Figure 3 presents the N_2_-sorption isotherms of RH-based porous materials where volume absorbance has been plotted against relative pressure. Changes in volume absorbance were presented with the relative pressure change.

The surface area of the samples of KOH-RH, AgNP–KOH-RH, and KOH-RHH presented 5.42, 13.57, and 27.87 m^2^/g, respectively. In an earlier study, untreated rice husk and hydrochar showed surface areas of 3.5 and 5.02 m^2^/g, respectively^70^. In this study, KOH treatment of untreated rice husk and hydrochar enhanced the surface area of both samples significantly. The incorporation of AgNPs and calcination increased the surface area by almost 10 m^2^/g for AgNP–KOH-RH. Moreover, the magnetic nanoparticles have strong magnetic interactions and could easily be aggregated together^71^.

#### Zeta particle size

**Figure 4:**
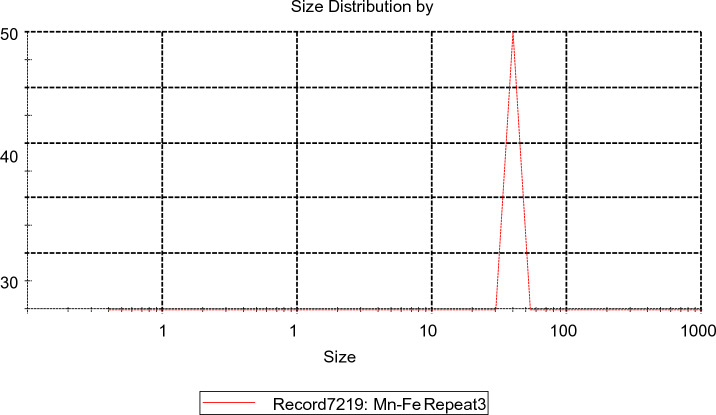
Zeta potential average particle size.

The Zeta Sizer Nano Series (ATA Scientific Pty Ltd., Australia) analyzed particle size and surface charge. 0.01 g of each sample was dispersed into 1 ml and 0.75 ml water separately into the different vessels for particle size and zeta potential (surface charge). Both cuvettes were inserted into the zeta sizer machine, and analyzed the results through Malvern software connected with the zeta sizer. The zeta potential of Fe–Mn bimetallic nanoparticles was observed at room temperature. The average size of nanoparticles was found at 39.8 nm, as shown in Fig. 4.

### Effect of adsorbent dosage

**Figure 5:**
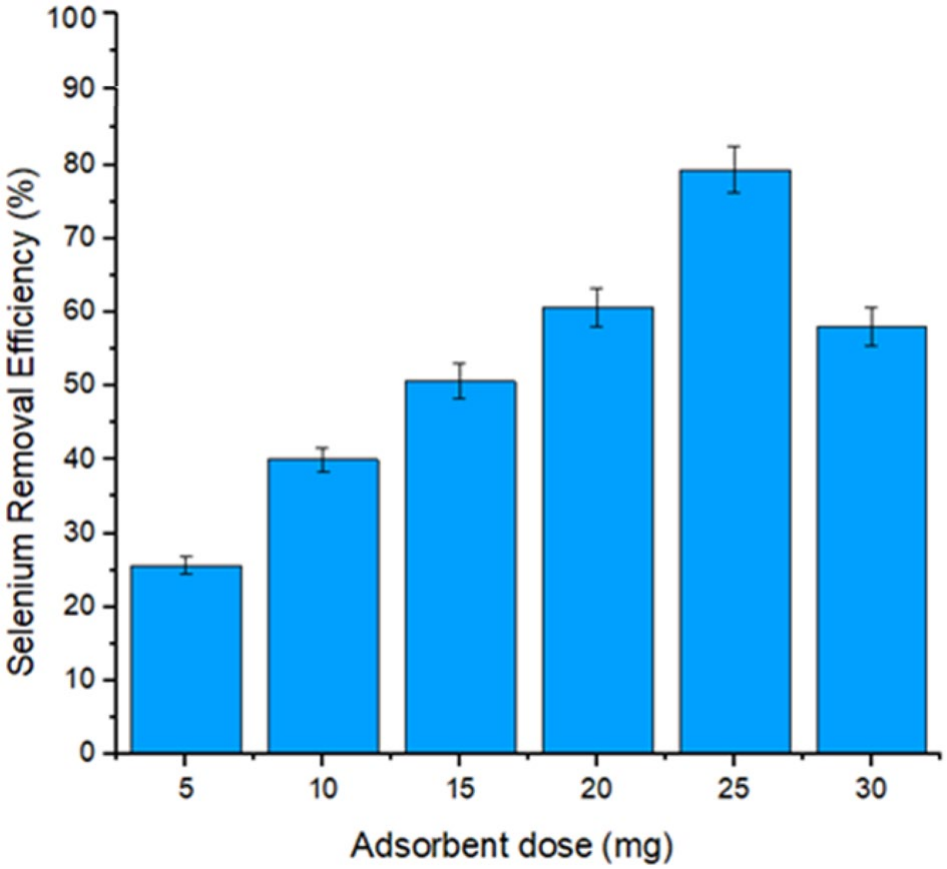
Effect of Adsorbent Dose on selenium Removal (%).

The effect of adsorbent dosage was the first parameter to determine the optimized condition because further examinations of selenium were dependent on this parameter. It is also an important parameter in predicting the material economy and cost. It can be seen that the removal percentage of selenium was decreased by increasing the adsorbent dosage after 25 mg. In such cases, the shock of the particles may have occurred between the selenium particles and the micro-composite particle's adsorbent surface. The maximum removal efficiency at 25 mg of the micro-particles adsorbent dose is shown in Fig. 5. Małgorzata Szlachta observed the effect of Fe–Mn hydrous oxides dosage on the adsorption of Se (IV) and Se (VI) ions from the water medium. As the adsorbent concentration increased from 0.5 to 5 g/L, the efficiency of removing selenium oxyanion also increased. These results agreed with previous outcomes, which also showed that the alteration in the adsorption is mainly dependent on the presence of active binding sites on the adsorbent surface^72^.

Furthermore, our present results showed that Selenite was almost removed up to (96.2%) when the adsorbent of 4 g/L was applied to the water medium. Moreover, when the concentration dosage was increased to 4 to 5 g/L, a slight change in the adsorption of about (3%) was observed. Likewise, the selenate uptake initially slightly increased with the adsorbent dosage rate of 2 g/L, at the highest adsorbent dose up to 11.1%.

### Effect of time

**Figure 6:**
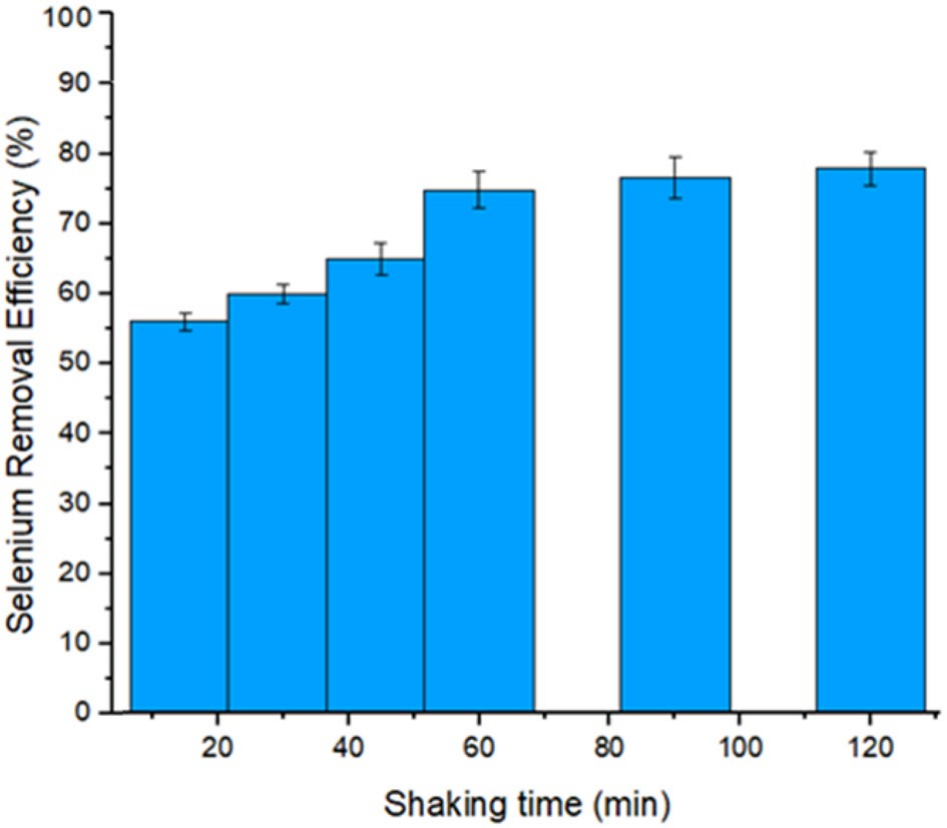
Effect of Shaking Time on selenium Removal (%).

The effect of shaking time is also an important parameter and assumes an essential function during the adsorption cycle^73^. It is observed from the result that by increasing the time of adsorption, the removal efficiency was also increased. The optimum selenium removal efficiency was observed at 79% after 60 min of shaking time, and then a slight increase in removal percentage became equilibrium when further contacted. This might be due to the saturation of all available adsorbent molecules, as shown in Fig. 6. The same effect was also observed in another study^61^. So, the optimum time was considered to be 60 min for further research.

Similarly, the previous literature reported other time-dependent Se(IV) adsorption mechanisms. Subsequently, Duc and his co-workers proposed that the adsorption efficiency gradually enhanced to a maximum level in the initial process. Moreover, it was also observed that when Se(IV) adsorbent was placed on the hematite surface, no significance was found between 4 and 65 h, respectively^74^.

Furthermore, the adsorption efficiency of selenite and selenate by BMDC as a function of time was conducted by Megha. It was noticed that the increase in sorption efficiency relates to a function of contact time^58^. Therefore, it was ameliorated that the fabricated bimetallic diatom composite represented strong Se(IV) uptake efficiency compared with Se(VI).

### Effect of pH

**Figure 7:**
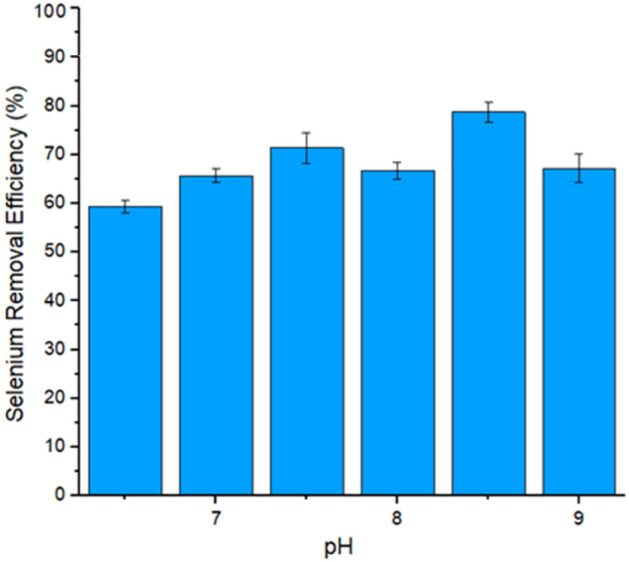
Effect of pH on selenium Removal (%).

Results showed that the removal efficiency of selenium towards bimetallic micro-composite adsorbent was higher (78.69%) at pH 8.5, which may be due to anionic SeO_2_ and positively charged adsorbent surface because surface charges of bimetallic micro-composite adsorbent are positive at pH 8.5 and negative at pH ≥ 9. It shows that an alkaline pH level increases the selenium ion adsorption efficiency^75^. Further increase in pH decreased the selenium adsorption efficiency towards bimetallic micro-composite adsorbent, as shown in Fig. 7. The effect of pH on final SeO_3_^2−^ concentration is presented in Fig. 7. The final Se concentration increased as the pH increased from 6.5 to 9.0, indicating that a lower pH favored Se adsorption. The better Se removal at lower pH could be an advantage for nano-magnetite use in treating mine water, which is typically low in pH^75^. When the pH was lower than 4.0, the SeO_3_^2−^ adsorption onto nano magnetite was relatively independent of pH. Consequently, the efficiency of the adsorbent was wide-ranging throughout the broad pH ranges, as reported in the previous results^19,76–78^. Though Se(IV) and Se(VI) adsorption efficiency were highly dependent on the pH factor, and as the pH concentration was increased towards the alkaline values, the removal efficacy steadily decreased. Therefore, at the adsorbed surface, the strong affinity for Se(IV) relative to Se(VI) significantly showed strong selenite adsorption relative to any given pH concentration^72^.

### Effect of adsorbate concentration

**Figure 8:**
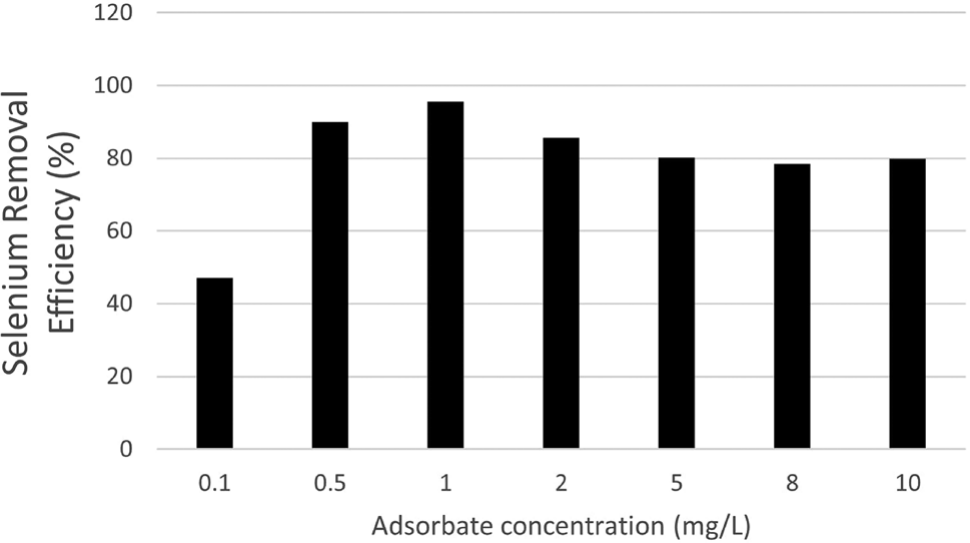
Effect of selenium Concentration Removal (%).

The effect of adsorbate concentration was determined by taking different amounts of selenium concentration ranging from 0.1 to 10 mg/L with an adsorbent dosage of 0.5 g/L and at pH 8.5 were stirred for 60 min on a horizontal orbital rotational shaker for accessing the effectiveness of selenium removal effectiveness. It can be seen from the result that an increase in the initial selenium concentration results in better removal of selenium up to 1 ppm of selenium solution. This was because of the expansion in the number of active sites accessible for the adsorption of selenium ions. Further increase in adsorbate dosage does not affect the removal efficiency of selenium due to a limited number of total available active sites, which became saturated at a higher concentration^62^. Treating the sample of 1 ppm, the selenium remains at 0.0546, and the maximum removal of 94.54% was achieved, as shown in Fig. 8. Another researcher examined the initial effect of Se concentration on the adsorption efficiency of iron oxide-impregnated CNTs. Generally, the observed outcomes proposed that as the initial concertation of Se increased, the adsorption efficiency also increased^61^. Previous literature also indicated that at initial Se concentrations between 5 and 20 ppm, the adsorption rate was sharp, so the adsorption process increased. At 20 ppm or above concentration, the adsorption rate decreases to a certain level. The strong adsorption efficiency at higher concentrations of Se is due to the increased mass transfer rate (driving forces) of selenium ions directed to the iron oxide-impregnated CNTs surfaces^79^. At the initial Se concentration of 40 ppm, the strong adsorption capacity was about 88 mg/g.

### Adsorption isotherms models

**Figure 9:**
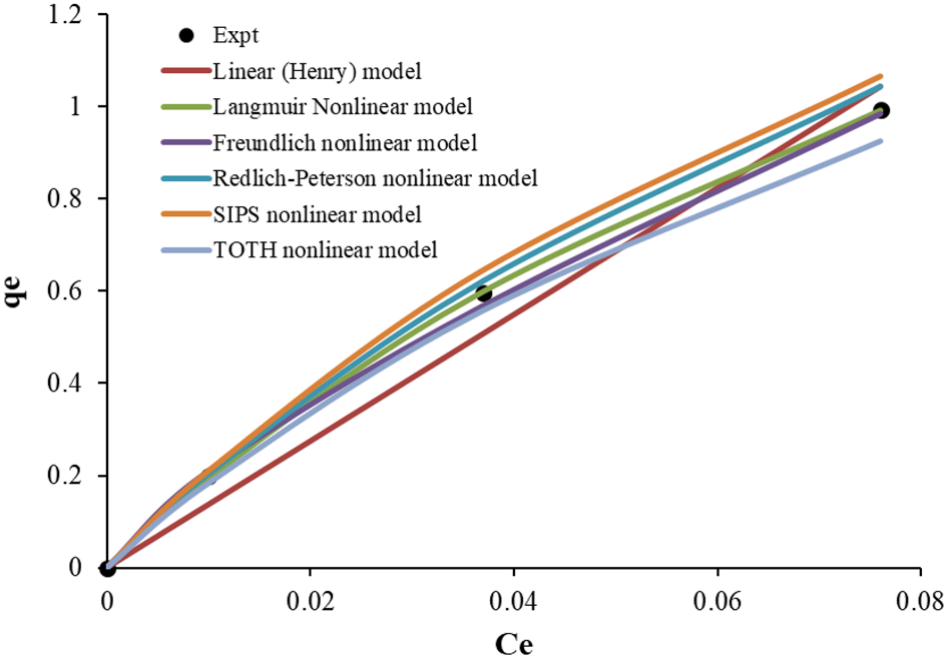
Comparison of performance of fitting different isotherm models.

The adsorption studies are verified against 6 prominent isotherm models as showin in Table 2. As stated earlier, most researchers linearize the non-linear model expressions. Even though the linearized model equation adequately fits the equilibrium data, providing higher R^2^, the predicted values give lower values after the isotherm parameters are substituted in the non-linear model form. Hence non-linear analysis is used to evaluate the model parameters without linearizing the non-linear expressions. The comparison of various isotherm parameters and different statistical metrics is given in Table 4. Among the validated isotherm models, Freundlich isotherm models seem to be more appropriate, resulting in an R^2^ value of 0.9961. Besides the highest R^2^, all other statistical metrics resulted in lower values. The comparison of the performance of different isotherm models investigated in this study is shown in Fig. 9. The best fitting of Freundlich isotherm model suggests that selenium adsorption took place heterogeneously due to the diversity of adsorption sites provided by bimetallic micro-particles.

**Table 4:** Comparison of various isotherm parameters along with different statistical metrics.

Nonlinear models	Parameters	R^2^	SSE	RMSE	χ^2^	HYBRID
Henry (Linear) model	K: 7.7	0.9894	2.7231	0.6237	1.4407	-32.0872
Langmuir model	KL: 0.213 qmax: 49.676	0.9927	1.7207	0.4958	0.3483	-12.6483
Freundlich model	KF: 8.531 n: 1.243	0.9961	0.9019	0.3530	0.0975	0.5275
Redlich-Peterson model	aRP: 7.075 KRP: 69.077 g: 0.224	0.9929	0.9359	0.3656	0.1001	0.1468
SIPS model	Ks: 0.042 qms: 212.776 ns: 0.831	0.9934	1.0408	0.3856	0.1073	-0.1377
TOTH model	aT: 3.826 KT: 2725.367 nT: 0.274	0.9930	1.1456	0.4045	0.1224	-2.5512

### Effect of reusability of micro composite

**Figure 10:**
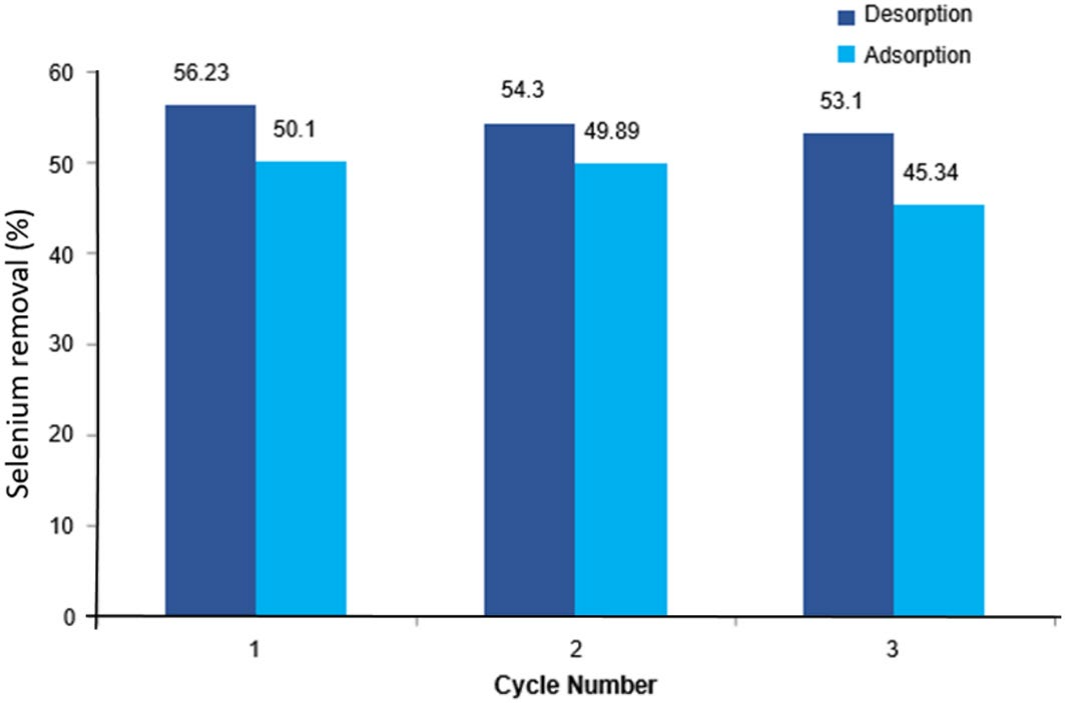
Effect of reusability of adsorbent on selenium remova.

From an industrial perspective, the regeneration ability of adsorbent materials is a crucial factor in estimating cost-effectiveness. The reusability of microparticles is very important in practical applications; therefore, bimetallic microparticles were used to test the reusability of selenium adsorption. Three successive adsorption/desorption cycles were performed to determine the reusability of particles. As a result, as shown in Fig. 10, desorption percentages gradually decrease from 56 to 53% from the first cycle to the third cycle. We presume that Se (IV) would preferably adsorb onto the high-energy sites with the strongest chemical bonding during the first removal cycle. Thus, the desorption percentage was slightly lower in the second and third regeneration cycles compared with the subsequent reuse cycles. However, the adsorption percentages decreased from the first to the third cycle. The adsorption percentage was around 50% in the first cycle and then reduced to 45% in the third.

## Conclusion

The outcomes of this study demonstrate the potential of iron-manganese bimetallic micro-composite adsorbents for eliminating selenium from water. Iron and manganese bimetallic microparticles were effectively created to remove more than 95% of selenium from water under the ideal conditions of pH- 8.5, 25 mg of adsorbent, 60 min of contact time, and an initial concentration of 1 mg/L, which resulted in the maximum selenium removal. A bimetallic micro-composite adsorbent had a 95% total selenium removal efficiency at optimal conditions. The Freundlich and Langmuir isotherms models were also used to analyze the equilibrium data. Both models had R^2^ values of (0.822) and (0.956), respectively. Instead of the Langmuir isotherm, equilibrium data effectively fit the Freundlich isotherm equation. The outcomes showed that iron and manganese bimetallic microparticles are the most promising candidate for removing selenium from wastewater.

## Data Availability

The datasets used and analyzed during the current study are available from the corresponding author upon reasonable request.

## References

[1] Martin, P. *et al.* Pakistan strategic country environmental assessment. *South Asia Region World Bank* **1**, 1–80 (2006).

[2] Rogers, P. & Hall, A. W. *Effective Water Governance* Vol. 7 (Global Water Partnership, 2003).

[3] Vinceti, M. *et al.* Cancer mortality in a residential cohort exposed to environmental selenium through drinking water. *J. Clin. Epidemiol.* **48**(9), 1091–1097 (1995). 7636510 10.1016/0895-4356(95)00014-u

[4] Etteieb, S., Magdouli, S., Zolfaghari, M. & Brar, S. Monitoring and analysis of selenium as an emerging contaminant in mining industry: A critical review. *Sci. Total Environ.* **698**, 134339 (2020). 31783461 10.1016/j.scitotenv.2019.134339

[5] Santos, S., Ungureanu, G., Boaventura, R. & Botelho, C. Selenium contaminated waters: an overview of analytical methods, treatment options and recent advances in sorption methods. *Sci. Total Environ.* **521**, 246–260 (2015). 25847169 10.1016/j.scitotenv.2015.03.107

[6] Fu, Y., Wang, J., Liu, Q. & Zeng, H. Water-dispersible magnetic nanoparticle–graphene oxide composites for selenium removal. *Carbon* **77**, 710–721 (2014).

[7] Awual, M. R., Yaita, T., Suzuki, S. & Shiwaku, H. Ultimate selenium (IV) monitoring and removal from water using a new class of organic ligand based composite adsorbent. *J. Hazard. Mater.* **291**, 111–119 (2015). 25771216 10.1016/j.jhazmat.2015.02.066

[8] Edition, F. Guidelines for drinking-water quality. *WHO Chronicle* **38**(4), 104–108 (2011). 6485306

[9] Awual, M. R., Hasan, M. M. & Khaleque, M. A. Efficient selenium (IV) detection and removal from water by tailor-made novel conjugate adsorbent. *Sens. Actuatoes B* **209**, 194–202 (2015).

[10] Awual, M. R. Efficient phosphate removal from water for controlling eutrophication using novel composite adsorbent. *J. Clean. Prod.* **228**, 1311–1319 (2019).

[11] Hasan, M. N., Shenashen, M., Hasan, M. M., Znad, H. & Awual, M. R. Assessing of cesium removal from wastewater using functionalized wood cellulosic adsorbent. *Chemosphere* **270**, 128668 (2021). 33268087 10.1016/j.chemosphere.2020.128668

[12] Awual, M. R. Novel ligand functionalized composite material for efficient copper (II) capturing from wastewater sample. *Compos. B Eng.* **172**, 387–396 (2019).

[13] Yamani, J. S., Lounsbury, A. W. & Zimmerman, J. B. Adsorption of selenite and selenate by nanocrystalline aluminum oxide, neat and impregnated in chitosan beads. *Water Res.* **50**, 373–381 (2014). 24238738 10.1016/j.watres.2013.10.054

[14] Gonzalez, C. M. *et al.* Sorption kinetic study of selenite and selenate onto a high and low pressure aged iron oxide nanomaterial. *J. Hazard. Mater.* **211**, 138–145 (2012). 21907486 10.1016/j.jhazmat.2011.08.023 PMC4338001

[15] Awual, M. R. A novel facial composite adsorbent for enhanced copper (II) detection and removal from wastewater. *Chem. Eng. J.* **266**, 368–375 (2015).

[16] Awual, M. R. *et al.* Radioactive cesium removal from nuclear wastewater by novel inorganic and conjugate adsorbents. *Chem. Eng. J.* **242**, 127–135 (2014).

[17] Martinez, M., Giménez, J., De Pablo, J., Rovira, M. & Duro, L. Sorption of selenium (IV) and selenium (VI) onto magnetite. *Appl. Surf. Sci.* **252**(10), 3767–3773 (2006).

[18] Rovira, M. *et al.* Sorption of selenium (IV) and selenium (VI) onto natural iron oxides: goethite and hematite. *J. Hazard. Mater.* **150**(2), 279–284 (2008). 17531378 10.1016/j.jhazmat.2007.04.098

[19] Chan, Y. T., Kuan, W. H., Chen, T. Y. & Wang, M. K. Adsorption mechanism of selenate and selenite on the binary oxide systems. *Water Res.* **43**(17), 4412–4420 (2009). 19628244 10.1016/j.watres.2009.06.056

[20] Tuzen, M. & Sarı, A. Biosorption of selenium from aqueous solution by green algae (Cladophora hutchinsiae) biomass: equilibrium, thermodynamic and kinetic studies. *Chem. Eng. J.* **158**(2), 200–206 (2010).

[21] Kongsri, S., Janpradit, K., Buapa, K., Techawongstien, S. & Chanthai, S. Nanocrystalline hydroxyapatite from fish scale waste: Preparation, characterization and application for selenium adsorption in aqueous solution. *Chem. Eng. J.* **215**, 522–532 (2013).

[22] Zhang, N., Lin, L.-S. & Gang, D. Adsorptive selenite removal from water using iron-coated GAC adsorbents. *Water Res.* **42**(14), 3809–3816 (2008). 18694584 10.1016/j.watres.2008.07.025

[23] Munjur, H. M. *et al.* Biodegradable natural carbohydrate polymeric sustainable adsorbents for efficient toxic dye removal from wastewater. *J. Mol. Liq.* **319**, 114356 (2020).

[24] Awual, M. R. New type mesoporous conjugate material for selective optical copper (II) ions monitoring & removal from polluted waters. *Chem. Eng. J.* **307**, 85–94 (2017).

[25] Balistrieri, L. S. & Chao, T. Selenium adsorption by goethite. *Soil Sci. Soc. Am. J.* **51**(5), 1145–1151 (1987).

[26] Balistrieri, L. S. & Chao, T. Adsorption of selenium by amorphous iron oxyhydroxide and manganese dioxide. *Geochim. Cosmochim. Acta* **54**(3), 739–751 (1990).

[27] Kuan, W.-H., Lo, S.-L., Wang, M. K. & Lin, C.-F. Removal of Se (IV) and Se (VI) from water by aluminum-oxide-coated sand. *Water Res.* **32**(3), 915–923 (1998).

[28] Mavrov, V., Stamenov, S., Todorova, E., Chmiel, H. & Erwe, T. New hybrid electrocoagulation membrane process for removing selenium from industrial wastewater. *Desalination* **201**(1–3), 290–296 (2006).

[29] Peak, D. Adsorption mechanisms of selenium oxyanions at the aluminum oxide/water interface. *J. Colloid Interface Sci.* **303**(2), 337–345 (2006). 16949599 10.1016/j.jcis.2006.08.014

[30] El-Shafey, E. Sorption of Cd (II) and Se (IV) from aqueous solution using modified rice husk. *J. Hazard. Mater.* **147**(1–2), 546–555 (2007). 17306927 10.1016/j.jhazmat.2007.01.051

[31] Hu, J., Lo, I. & Chen, G. Removal of Cr (VI) by magnetite. *Water Sci. Technol.* **50**(12), 139–146 (2004). 15686014

[32] Hu, J., Chen, G. & Lo, I. M. Removal and recovery of Cr (VI) from wastewater by maghemite nanoparticles. *Water Res.* **39**(18), 4528–4536 (2005). 16146639 10.1016/j.watres.2005.05.051

[33] Hu, J., Lo, I. M. & Chen, G. Performance and mechanism of chromate (VI) adsorption by δ-FeOOH-coated maghemite (γ-Fe2O3) nanoparticles. *Sep. Purif. Technol.* **58**(1), 76–82 (2007).

[34] Lazaridis, N., Bakoyannakis, D. & Deliyanni, E. Chromium (VI) sorptive removal from aqueous solutions by nanocrystalline akaganeite. *Chemosphere* **58**(1), 65–73 (2005). 15522334 10.1016/j.chemosphere.2004.09.007

[35] Yuan, P. *et al.* Removal of hexavalent chromium [Cr (VI)] from aqueous solutions by the diatomite-supported/unsupported magnetite nanoparticles. *J. Hazard. Mater.* **173**(1–3), 614–621 (2010). 19748178 10.1016/j.jhazmat.2009.08.129

[36] Awual, M. R., Hasan, M. M., Ihara, T. & Yaita, T. Mesoporous silica based novel conjugate adsorbent for efficient selenium (IV) detection and removal from water. *Microporous Mesoporous Mater.* **197**, 331–338 (2014).

[37] Awual, M. R., Hasan, M. M. & Khaleque, M. A. Efficient selenium (IV) detection and removal from water by tailor-made novel conjugate adsorbent. *Sens. Actuators B* **209**, 194–202 (2015).

[38] Jiménez-Cedillo, M., Olguín, M., Fall, C. & Colín, A. Adsorption capacity of iron-or iron–manganese-modified zeolite-rich tuffs for As (III) and As (V) water pollutants. *Appl. Clay Sci.* **54**(3–4), 206–216 (2011).

[39] Liu, W., Sutton, N. B., Rijnaarts, H. H. & Langenhoff, A. A. Pharmaceutical removal from water with iron-or manganese-based technologies: A review. *Crit. Rev. Environ. Sci. Technol.* **46**(19–20), 1584–1621 (2016).

[40] Awual, M. R. Mesoporous composite material for efficient lead (II) detection and removal from aqueous media. *J. Environ. Chem. Eng.* **7**(3), 103124 (2019).

[41] Ghosh, P. K. Hexavalent chromium [Cr (VI)] removal by acid modified waste activated carbons. *J. Hazard. Mater.* **171**(1–3), 116–122 (2009). 19553008 10.1016/j.jhazmat.2009.05.121

[42] Granados-Correa, F. & Bulbulian, S. Co (II) adsorption in aqueous media by a synthetic Fe–Mn binary oxide adsorbent. *Water Air Soil Pollut.* **223**(7), 4089–4100 (2012).

[43] Mamais, D. *et al.* Biological groundwater treatment for chromium removal at low hexavalent chromium concentrations. *Chemosphere* **152**, 238–244 (2016). 26971177 10.1016/j.chemosphere.2016.02.124

[44] Islam, M. A., Angove, M. J., Morton, D. W., Pramanik, B. K. & Awual, M. R. A mechanistic approach of chromium (VI) adsorption onto manganese oxides and boehmite. *J. Environ. Chem. Eng.* **8**(2), 103515 (2020).

[45] Manning, B. A., Kiser, J. R., Kwon, H. & Kanel, S. R. Spectroscopic investigation of Cr (III)-and Cr (VI)-treated nanoscale zerovalent iron. *Environ. Sci. Technol.* **41**(2), 586–592 (2007). 17310726 10.1021/es061721m

[46] Ludwig, R. D. *et al.* In situ chemical reduction of Cr (VI) in groundwater using a combination of ferrous sulfate and sodium dithionite: A field investigation. *Environ. Sci. Technol.* **41**(15), 5299–5305 (2007). 17822094 10.1021/es070025z

[47] Zhu, J. *et al.* Adsorption behavior and removal mechanism of arsenic on graphene modified by iron–manganese binary oxide (FeMnO x/RGO) from aqueous solutions. *RSC Adv.* **5**(83), 67951–67961 (2015).

[48] Singh, N. H., Kezo, K., Debnath, A. & Saha, B. Enhanced adsorption performance of a novel Fe-Mn-Zr metal oxide nanocomposite adsorbent for anionic dyes from binary dye mix: Response surface optimization and neural network modeling. *Appl. Organomet. Chem.* **32**(3), e4165 (2018).

[49] Hu, J., Lo, I. M. & Chen, G. Fast removal and recovery of Cr (VI) using surface-modified jacobsite (MnFe2O4) nanoparticles. *Langmuir* **21**(24), 11173–11179 (2005). 16285787 10.1021/la051076h

[50] Deschamps, E., Ciminelli, V. S. & Höll, W. H. Removal of As (III) and As (V) from water using a natural Fe and Mn enriched sample. *Water Res.* **39**(20), 5212–5220 (2005). 16290184 10.1016/j.watres.2005.10.007

[51] Zhang, G., Qu, J., Liu, H., Liu, R. & Wu, R. Preparation and evaluation of a novel Fe–Mn binary oxide adsorbent for effective arsenite removal. *Water Res.* **41**(9), 1921–1928 (2007). 17382991 10.1016/j.watres.2007.02.009

[52] Lou, Z. *et al.* Enhanced removal of As (III)/(V) from water by simultaneously supported and stabilized Fe-Mn binary oxide nanohybrids. *Chem. Eng. J.* **322**, 710–721 (2017).

[53] Ning, Q. *et al.* Fabrication of hydrochar functionalized Fe–Mn binary oxide nanocomposites: characterization and 17β-estradiol removal. *RSC Adv.* **7**(59), 37122–37129 (2017).

[54] El-Mehalmey, W. A. *et al.* Metal–organic framework@ silica as a stationary phase sorbent for rapid and cost-effective removal of hexavalent chromium. *J. Mater. Chem. A* **6**(6), 2742–2751 (2018).

[55] Zhu, S., Huang, X., Wang, D., Wang, L. & Ma, F. Enhanced hexavalent chromium removal performance and stabilization by magnetic iron nanoparticles assisted biochar in aqueous solution: Mechanisms and application potential. *Chemosphere* **207**, 50–59 (2018). 29772424 10.1016/j.chemosphere.2018.05.046

[56] Zhang, X., Dong, J., Hao, Z., Cai, W. & Wang, F. Fe–Mn/MCM-41: Preparation, characterization, and catalytic activity for methyl orange in the process of heterogeneous fenton reaction. *Trans. Tianjin Univ.* **24**(4), 361–369 (2018).

[57] Yuan, Z., Cheng, X., Zhong, L., Wu, R. & Zheng, Y. Preparation, characterization and performance of an electrospun carbon nanofiber mat applied in hexavalent chromium removal from aqueous solution. *J. Environ. Sci.* **77**, 75–84 (2019). 10.1016/j.jes.2018.06.016 30573108

[58] Thakkar, M. & Mitra, S. Bimetallic oxide nanohybrid synthesized from diatom frustules for the removal of selenium from water. *J. Nanomater.* **2017**, 1–9 (2017).

[59] Chen, M.-L. & An, M.-I. Selenium adsorption and speciation with Mg–FeCO3 layered double hydroxides loaded cellulose fibre. *Talanta* **95**, 31–35 (2012). 22748552 10.1016/j.talanta.2012.03.038

[60] Ma, Z., Shan, C., Liang, J. & Tong, M. Efficient adsorption of Selenium (IV) from water by hematite modified magnetic nanoparticles. *Chemosphere* **193**, 134–141 (2018). 29131972 10.1016/j.chemosphere.2017.11.005

[61] Bakather, O. Y., Kayvani Fard, A., Khraisheh, M., Nasser, M. S. & Atieh, M. A. Enhanced adsorption of selenium ions from aqueous solution using iron oxide impregnated carbon nanotubes. *Bioinorg. Chem. Appl.* **2017**, 1–12 (2017). 10.1155/2017/4323619 PMC5438866 28555093

[62] Zelmanov, G. & Semiat, R. Selenium removal from water and its recovery using iron (Fe3+) oxide/hydroxide-based nanoparticles sol (NanoFe) as an adsorbent. *Sep. Purif. Technol.* **103**, 167–172 (2013).

[63] Bleiman, N. & Mishael, Y. G. Selenium removal from drinking water by adsorption to chitosan–clay composites and oxides: Batch and columns tests. *J. Hazard. Mater.* **183**(1–3), 590–595 (2010). 20708334 10.1016/j.jhazmat.2010.07.065

[64] Bax, D., Peters, F., Van Noort, J. & Agterdenbos, J. The determination of selenium with hydride generation AAS—II: The role of sodium borohydride and of hydrogen gas. *Spectrochim. Acta Part B* **41**(3), 275–282 (1986).

[65] Dehghani, M. H. *et al.* Statistical modelling of endocrine disrupting compounds adsorption onto activated carbon prepared from wood using CCD-RSM and DE hybrid evolutionary optimization framework: Comparison of linear vs non-linear isotherm and kinetic parameters. *J. Mol. Liq.* **302**, 112526 (2020).

[66] Karri, R. R., Sahu, J. N. & Jayakumar, N. S. Optimal isotherm parameters for phenol adsorption from aqueous solutions onto coconut shell based activated carbon: Error analysis of linear and non-linear methods. *J. Taiwan Inst. Chem. Eng.* **80**, 472–487 (2017).

[67] Beesley, L. & Marmiroli, M. The immobilisation and retention of soluble arsenic, cadmium and zinc by biochar. *Environ. Pollut.* **159**(2), 474–480 (2011). 21109337 10.1016/j.envpol.2010.10.016

[68] Islam, A., Teo, S. H., Awual, M. R. & Taufiq-Yap, Y. H. Assessment of clean H2 energy production from water using novel silicon photocatalyst. *J. Clean. Prod.* **244**, 118805 (2020).

[69] Ullrich, A., Rahman, M. M., Longo, P. & Horn, S. Synthesis and high-resolution structural and chemical analysis of iron-manganese-oxide core-shell nanocubes. *Sci. Rep.* **9**(1), 1–9 (2019). 31848357 10.1038/s41598-019-55397-z PMC6917765

[70] Hossain, N. *et al.* Synthesis and characterization of rice husk biochar via hydrothermal carbonization for wastewater treatment and biofuel production. *Sci. Rep.* **10**(1), 18851 (2020). 33139793 10.1038/s41598-020-75936-3 PMC7606520

[71] Nandi, D. *et al.* Manganese-incorporated iron (III) oxide–graphene magnetic nanocomposite: Synthesis, characterization, and application for the arsenic (III)-sorption from aqueous solution. In *Nanotechnology for Sustainable Development* 149–162 (Springer, 2012).

[72] Szlachta, M. & Chubar, N. The application of Fe–Mn hydrous oxides based adsorbent for removing selenium species from water. *Chem. Eng. J.* **217**, 159–168 (2013).

[73] Kubra, K. T. *et al.* Sustainable detection and capturing of cerium (III) using ligand embedded solid-state conjugate adsorbent. *J. Mol. Liq.* **338**, 116667 (2021).

[74] Duc, M., Lefevre, G. & Fédoroff, M. Sorption of selenite ions on hematite. *J. Colloid Interface Sci.* **298**(2), 556–563 (2006). 16483595 10.1016/j.jcis.2006.01.029

[75] Wei, X., Bhojappa, S., Lin, L.-S. & Viadero, R. C. Jr. Performance of nano-magnetite for removal of selenium from aqueous solutions. *Environ. Eng. Sci.* **29**(6), 526–532 (2012).

[76] Gonzalez, C. M., Hernandez, J., Parsons, J. G. & Gardea-Torresdey, J. L. A study of the removal of selenite and selenate from aqueous solutions using a magnetic iron/manganese oxide nanomaterial and ICP-MS. *Microchem. J.* **96**(2), 324–329 (2010).

[77] Jordan, N. *et al.* Sorption of selenium (VI) onto anatase: Macroscopic and microscopic characterization. *Geochim. Cosmochim. Acta* **75**(6), 1519–1530 (2011).

[78] Awual, M. R. *et al.* Selective cesium removal from radioactive liquid waste by crown ether immobilized new class conjugate adsorbent. *J. Hazard. Mater.* **278**, 227–235 (2014). 24981675 10.1016/j.jhazmat.2014.06.011

[79] Lounsbury, A. W., Yamani, J. S., Johnston, C. P., Larese-Casanova, P. & Zimmerman, J. B. The role of counter ions in nano-hematite synthesis: Implications for surface area and selenium adsorption capacity. *J. Hazard. Mater.* **310**, 117–124 (2016). 26905609 10.1016/j.jhazmat.2016.01.078

