# Author Correction: Rapid adsorption of selenium removal using iron manganese-based micro adsorbent

**DOI:** 10.1038/s41598-025-86902-2

**Published:** 2025-01-28

**Authors:** Sundus Saeed Qureshi, Sheeraz Ahmed Memon, Rafi-ul-Zaman, Nanik Ram, Sumbul Saeed, Nabisab Mujawar Mubarak, Rama Rao Karri

**Affiliations:** 1 Institute of Environmental Engineering and Management, Mehran University of Engineering and Technology, Jamshoro, 76090 Sindh Pakistan; 2 College of Plant Sciences and Technology, Huazhong Agricultural University, Wuhan, 430070 People’s Republic of China; 3 Petroleum and Chemical Engineering, Faculty of Engineering, Universiti Teknologi Brunei, Bandar Seri Begawan, 1410 Brunei Darussalam

Correction to: *Scientific Reports* 10.1038/s41598-022-21275-4, published online 14 October 2022

The original version of this Article contained errors. Due to translation errors some non-standard phrases and incorrect wording have been used. In addition, during manuscript preparation lower pH-values have been included in the text, which are not presented in the figures.

In the Material and methods section, under the subheading ‘Synthesis of iron-manganese (Fe–Mn) bimetallic micro-particles’,

“The arrangement of salts was set on the attractive stirrer for proper mixing.”

now reads:

“The arrangement of salts was set on the magnetic stirrer for proper mixing.”

Under the subheading ‘Batch adsorption experiments’,

“All the readings were completed in 50 ml answers for the selenium removal from water.”

now reads:

“All the readings were completed in 50 ml arrangements for the selenium removal from water.”

Further,

 “The impact of shaking time on the efficiency of selenium removal by bimetallic micro-composite adsorbent was studied by taking all the other parameters constant with a solution of the initial concentration of 10 ppm, 0.5 g/L of adsorbent dosage at 4 pH of the solution by varying time from 15 to 120 min.”

now reads:

“The impact of shaking time on the efficiency of selenium removal by bimetallic micro-composite adsorbent was studied by taking all the other parameters constant with a solution of the initial concentration of 10 ppm, 0.5 g/L of adsorbent dosage at 6.5 pH of the solution by varying time from 15 to 120 min.”

And,

“The material cost was studied on six different dosages from 5 to 30 mg while keeping the range of various boundaries steady, i.e., 10 ppm selenium arrangement at pH 4 fomented for 1 h for finding the selenium removal efficiency and removal limit of bimetallic micro-composite adsorbent.”

now reads:

“The selenium removal efficiency was studied on six different dosages from 5 to 30 mg while keeping the range of various boundaries steady, i.e., 10 ppm selenium arrangement at pH 6.5 fomented for 1 h for finding the selenium removal efficiency and removal limit of bimetallic micro-composite adsorbent.”

In the Results and discussion section, under the subheading ‘Scanning electron microscope (SEM) analysis’,

“The SEM images of the different focus reveal that microparticles have been synthesized successfully and have the fine granular structure been spherical.”

now reads:

“The SEM images reveal that microparticles have been synthesized successfully and have the fine granular structure been spherical.”

Under the subheading ‘Effect of pH’,

“The final Se concentration increased as the pH increased from 4.0 to 9.0, indicating that a lower pH favored Se adsorption.”

now reads:

“The final Se concentration increased as the pH increased from 6.5 to 9.0, indicating that a lower pH favored Se adsorption.”

Finally, due to an error during figure preparation, the unit description of the x-axis in Figure 8 was incorrect. Instead of ‘mg’ the correct unit is ‘w/v (mg/L)’. The original Figure 8 and accompanying legend appear below.

**Fig. 8:**
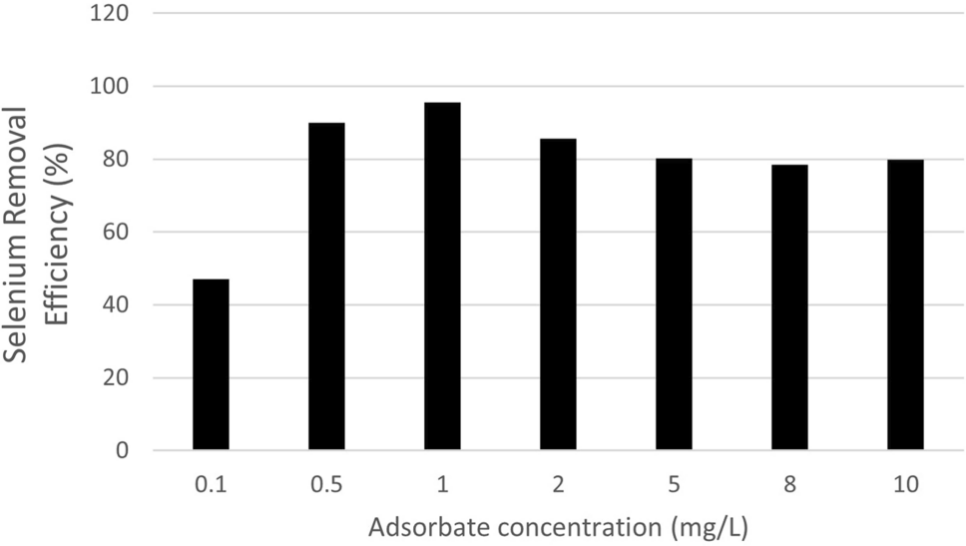
Effect of adsorbate concentration on selenium removal (%).

The original Article has been corrected.

